# A non-neuronal fMRI signal: both a confound and an opportunity for insight

**DOI:** 10.1038/s41386-026-02512-0

**Published:** 2026-08-04

**Authors:** Amy C. Janes, Cole Korponay, Tianye Zhai, Blaise B. Frederick

**Affiliations:** 1 Neuroimaging Research Branch, Intramural Research Program, National Institute on Drug Abuse, National Institutes of Health, Baltimore, MD USA; 2 Department of Psychiatry, Harvard University Medical School, Boston, MA USA; 3 McLean Hospital Brain Imaging Center, Belmont, MA USA

**Keywords:** Neuroscience, Medical research

## Abstract

Functional magnetic resonance imaging (fMRI) is widely used to investigate brain function. However, interpretation of the blood oxygen level-dependent (BOLD) signal is complicated by the fact that it reflects both neuronal activity and vascular physiology. This problem is especially relevant in neuropsychopharmacology research because pharmacological and psychiatric effects on brain function are often accompanied by physiological shifts. Here, we discuss recent evidence for an approach to disentangling neuronal and physiological components of the BOLD signal that enhances the interpretability and clinical utility of fMRI. Converging findings suggest that the systemic low-frequency oscillation (sLFO), a major component of the global signal, indexes cardiovascular manifestations of arousal rather than neuronal function. As such, retaining the sLFO in fMRI data can substantially distort functional connectivity estimates, leading to the misinterpretation of physiological fluctuations in arousal as neuronal effects. At the same time, the sLFO itself tracks physiological arousal level, behavioral performance, drug craving, pharmacological modulation, and large-scale brain network organization. Collectively, we suggest that when interpreting fMRI data, sLFO-indexed physiological arousal should both be modeled and considered separately, as extracting this signal from fMRI data provides dual benefits: a cleaner neuronal signal and a complementary index of behaviorally relevant physiology.

Functional magnetic resonance imaging (fMRI) is widely used to investigate brain function. However, interpretation of the blood oxygen level-dependent (BOLD)-derived signal is complicated by the fact that it reflects not only neuronal activity, through neurovascular coupling, but also other vascular and physiological processes. This issue is particularly important in neuropsychopharmacology, where psychiatric states and pharmacological interventions alter physiological arousal alongside neuronal signaling, complicating the interpretation of observed changes in fMRI measures.

The tension between neuronal interpretation and physiological contribution is apparent in debates surrounding the treatment of the global signal. Global signal regression (GSR) remains controversial because it can introduce spurious anticorrelations in functional connectivity. At the same time, the global signal is increasingly recognized as reflecting biologically meaningful variation related to physiological arousal rather than nuisance variance alone [1,2]. As a result, some are hesitant to remove the global signal not only because of potential distorted connectivity estimates, but because the signal may contain meaningful biological information.

We address these concerns and discuss how this signal is linked to clinically relevant states and large-scale brain network function. We argue that *dynamic* GSR (dGSR), which accounts for blood arrival time, improves the specificity of functional connectivity by reducing physiologically driven influences in the BOLD data without introducing known issues due to traditional *static* GSR. In support of this, recent benchmarking across resting-state and task fMRI demonstrates that dGSR improves denoising performance relative to current preprocessing methods [3]. The dGSR approach (https://github.com/bbfrederick/rapidtide) specifically identifies the systemic low-frequency oscillation (sLFO; ~0.01–0.15 Hz), a major component of the global signal, that is generated in the periphery and propagates through the brain via blood flow. Although additional non-neuronal sources of variance likely contribute to the BOLD signal, the present Perspective focuses on the sLFO because of its established physiological origin and implications for neuropsychopharmacology. The sLFO measured in brain-based BOLD data is strongly correlated with the sLFO observed in peripheral sites such as the fingertip, supporting its non-neuronal systemic origin [4]. Although neuronal signals occupy the same low-frequency band, the sLFO can be separated from components reflecting local neuronal activity, thereby improving the neuronal specificity of subsequent analyses. Importantly, this physiological signal is not merely nuisance variance that can potentially confound fMRI findings but provides an index of physiological arousal [1,2].

Across multiple independent datasets, the sLFO systematically increases during fMRI scans as arousal decreases, as assessed by changes in heart rate variability, pupil diameter, and respiration [2]. These findings demonstrate that arousal-related physiological signals are embedded within fMRI. Critically, this increase in sLFO over time drives a substantial, artifactual rise in whole-brain functional connectivity, distorting the spatial and temporal structure of connectivity estimates. While the sLFO indexes physiological arousal, strong evidence that it is primarily driven by cardiovascular processes indicates that it does not directly reflect neuronal activity. Consequently, retaining this confound may lead to physiological fluctuations in arousal being misinterpreted as neuronally derived changes in functional connectivity. This issue is particularly relevant in studies where physiological arousal may vary, including investigations of dynamic brain properties, comparisons across clinical populations, evoking emotional states, and pharmacological manipulations.

Further, the sLFO appears to reflect physiologically meaningful variation that is linked to behavior and clinically relevant states. Consistent with the known arousal-enhancing effects of the dopamine and norepinephrine agonist methylphenidate, an acute dose of methylphenidate significantly reduced the sLFO, concordant with the sLFO’s inverse relationship with arousal [5]. This reduction was associated with faster behavioral reaction times, linking sLFO-derived measures of physiology to expected behavior. Similarly, in individuals with nicotine dependence, exposure to emotionally evocative smoking cues reduced the sLFO [5], fitting with cue-induced increases in arousal. On average, this cue-induced reduction in the sLFO diminished over repeated cue exposure, but individuals who exhibited less habituation reported greater cue-induced craving, linking more sustained physiological arousal to heightened subjective craving. These findings suggest that the sLFO captures clinically relevant changes in physiological arousal across both pharmacological and task-based manipulations. Consequently, some effects commonly interpreted as reflecting neural modulation in fMRI may also be influenced by physiological changes in arousal.

These relationships extend beyond behavior and subjective experience to large-scale brain network function. Heightened arousal, indexed by lower sLFO amplitude, was associated with stronger anticorrelations between the default mode network (DMN) and the dorsal attention (DAN) and salience networks (SN), as well as altered temporal engagement of these systems [6]. These findings are notable given the established role of interactions among these large-scale networks in cognition and psychopathology. Importantly, these relationships persisted following sLFO denoising, suggesting that physiological arousal and neuronal network organization are distinct but related processes. Together, these findings suggest that physiological arousal may represent an important and previously underappreciated contributor to variability in functional connectivity and clinically relevant neuroimaging findings.

Moreover, pharmacological modulation of catecholaminergic function produces coordinated changes in both sLFO amplitude and network connectivity, even when connectivity is estimated following sLFO denosing. Methylphenidate-induced decreases in the sLFO, consistent with its arousal-enhancing effects, were associated with stronger anticorrelations between the DMN and both the DAN and SN. In contrast, under the dopamine D2/D3 antagonist haloperidol, drug-induced increases in the sLFO were associated with weaker anticorrelations among these networks. Together, these findings suggest that variability in functional connectivity among large-scale cognitive networks may substantially reflect shifts in physiological arousal in addition to neuronal signaling alone. More broadly, these observations raise the possibility that physiological arousal may substantially contribute to large-scale brain network organization, with important implications for how functional connectivity findings are interpreted in neuropsychopharmacology.

Collectively, these findings support a framework in which the sLFO, a major component of the global signal, is understood not simply as noise to be ignored, but as a biologically meaningful physiological signal that must be explicitly modeled and considered separately when interpreting fMRI data. Rather than diminishing the utility of fMRI, this framework improves the interpretability of neuroimaging by better separating neuronal and physiological contributions to the BOLD signal, yielding both more specific estimates of neuronal brain function and a physiological measure of arousal with behavioral, clinical, and neurobiological relevance.
